# Shear Bond Strength of Self-Adhesive Flowable Resin Composite

**DOI:** 10.1155/2022/6280624

**Published:** 2022-05-06

**Authors:** Norah Sibai, Aminah El Mourad, Nohair Al Suhaibani, Raghad Al Ahmadi, Sara Al Dosary

**Affiliations:** ^1^Division of Operative Dentistry, Department of Restorative Dental Sciences, College of Dentistry, King Saud University, P.O. Box 5967, Riyadh 11432, Saudi Arabia; ^2^Department of Pediatric Dentistry, King Saud Medical City, Ministry of Health, Riyadh 12746, Saudi Arabia; ^3^King Saud Medical City, Ministry of Health, Riyadh 12746, Saudi Arabia; ^4^Department of Oral Medicine and Diagnostic Science, College of Dentistry, King Saud University, P.O. Box 5967, Riyadh 11432, Saudi Arabia

## Abstract

This study aimed to evaluate the shear bond strength of self-adhesive flowable resin composite on both enamel and dentin and investigate whether surface pretreatment with a phosphoric acid etch affects the bond strength. In this in vitro study, 80 sound human premolars were flattened to create (40) uniform enamel (*E*) and (40) dentin (*D*) surfaces. Groups were divided according to surface pretreatment: E1 : Scotchbond^™^ Universal Etchant + Single Bond Universal Adhesive + Filtek^™^ Z350 XT flowable composite; E2: Single Bond Universal self-etch adhesive + Filtek Z350 XT flowable composite; E3 : Scotchbond Universal Etchant + Fusio Liquid Dentin self-adhesive flowable composite; E4: Fusio Liquid Dentin self-adhesive flowable composite; D1 : Scotchbond Universal Etchant + Single Bond Universal Adhesive + Filtek Z350 XT flowable composite; D2: Single Bond Universal self-etch adhesive + Filtek Z350 XT flowable composite; D3: Scotchbond Universal Etchant + Fusio Liquid Dentin self-adhesive flowable composite; D4: Fusio Liquid Dentin self-adhesive flowable composite. After 2500 thermal cycles, the SBS was measured with a universal testing machine. One-way analysis of variance and Tukey's test for multiple comparisons were used to compare results. Two-way ANOVA was done to observe the significance of interaction between the type of surface and treatment. The maximum (49.38 ± 1.23 MPa) and minimum (21.41 ± 5.27 MPa) SBS values were noted in groups D1 and E2, respectively. Shear bond test results showed that self-adhesive flowable composite exhibited similar shear bond strengths on enamel and dentin and the bond strength improved with selective acid etching.

## 1. Introduction

Esthetic dentistry is attracting increasing research attention in dental science. The use of adhesive resin composites has been confirmed as the best method for conservatively and esthetically restoring minimally-to-moderately cavitated teeth [1]. Flowable composites were first introduced in the mid-1990s [2]. Their smaller filler particle size and the lower filler content led to their lower viscosity and improved adaptability to cavity walls compared with more viscous conventional restorative composites [3–7]. They are currently recommended for small Class I and Class III lesions that are not under heavy occlusal stresses [8].

The advances in self-etch adhesive systems have been proposed to reduce the technique sensitivity, time consumption, and possibility of postoperative sensitivity of previously used etch-and-rinse adhesive systems. When a self-etching adhesive is applied, substrate demineralization and resin penetration occur simultaneously. These adhesives are known to perform well when used with dentin. However, the enamel porosity and the degree of resin penetration of these adhesives in enamel are causes for ongoing concern [9–11]. Many studies have shown that the strength of the bonds formed between dental composites and enamel using ultramild, mild, or moderately strong self-etch adhesive systems is lower than that of the bonds formed using etch-and-rinse adhesive systems [9–12]. Selective phosphoric acid etching of enamel has been suggested as a way of improving the bond strength of self-adhesive systems with respect to the enamel. Furthermore, acid pretreatment of the enamel surface can significantly increase the bond strength of one- and two-step self-etch adhesives [13, 14].

In recent years, dental companies have exploited the convenience of self-etch adhesives and the versatility of flowable composites to develop innovative composites that exhibit both self-adhesion and good flowability; some examples include Vertise^™^ Flow (Kerr Corporation, Orange, CA, USA) and Fusio^™^ Liquid Dentin (Pentron Clinical Technologies LLC, Orange, CA, USA). These materials exhibit self-adhesive behavior similar to that of self-etch adhesives [15–17]. Tuloglu et al. [18] reported that self-adhesive flowable composites have significantly lower failure rates as their use involves fewer clinical steps; specifically, the etching, priming, and bonding steps no longer have to be performed.

Since the introduction of self-adhesive flowable composites, several studies have evaluated their bond strength [15–19]. However, as these materials are fairly new to the market, their bond strength and clinical performance have not been evaluated in adequate detail. In this light, the present study aims to evaluate the shear bond strengths of self-adhesive flowable composite resins on enamel and dentin substrates and to determine whether phosphoric acid etch pretreatment of the enamel and dentin substrates affects the shear bond strength. Finally, the bond strength of the self-adhesive composite was compared to those of a self-etch adhesive and an etch-and-rinse adhesive used in conjunction with a conventional flowable nanocomposite.

### 1.1. The Tested Hypotheses

The tested hypotheses were as follows:

1. There is no significant difference in the shear bond strengths of self-adhesive flowable composite resin when bonded to enamel or dentin from those of self-etch and etch-and-rinse adhesives used with a conventional flowable nanocomposite
2. The shear bond strength of self-adhesive flowable composite is not affected by phosphoric acid etch pretreatment of the enamel and dentin substrates

## 2. Materials and Methods

This in vitro, comparative, experimental study used 80 sound human premolars extracted for orthodontic purposes. These premolars were first cleaned with an ultrasonic scaler and then polished with pumice using a slow-speed handpiece (No. 6412500, Kavo EWL, West Germany). Then, each tooth was examined individually under a stereomicroscope (Leica/Meyer Instruments, Houston, USA) to eliminate those with any defects. Teeth with restorations, caries, cracks, white-spot lesions, abrasion faucets, fluorosis, hypoplastic enamel, and extraction-related damage were excluded. The remaining teeth were kept at room temperature in a dark container filled with 0.05% thymol solution until mounting.

The tests specimens were prepared by separating the crowns 2 mm apical to the cementoenamel junction with the use of a slow-speed diamond saw and water cooling (Isomet 2000, Buehler, Illinois, USA). To mount the teeth, custom-made polyvinyl chloride cylindrical molds with a diameter of 34 mm and a height of 20 mm were filled with a self-curing acrylic resin. Each tooth was embedded in the acrylic resin with its buccal surface directed upward for bonding. The specimens were submerged in cold water to protect the teeth from the temperature rise due to acrylic resin polymerization.

After complete setting of the embedding resin, the buccal surfaces of the teeth were successively ground and polished using wet 240-, 400-, and 600-grit silicon carbide paper disks (Buehler, Illinois, USA) mounted on an AUTOMATA Pressair system (Jean Wirtz GMBH, Germany). This was done to create a uniform flat surface on the enamel (40 specimens) and dentin (40 specimens) samples that was flush with the mounting acrylic resin and was therefore suitable for bonding. The specimens were then divided randomly into eight groups of 10 teeth each for the different composite materials and surface pretreatment protocols used.  Table 1 lists the materials used in this study.

The test groups were as follows:

1. Enamel 1: enamel surface treated with Scotchbond^™^ Universal Etchant (3M ESPE, St Paul, MN, USA), Single Bond Universal Adhesive (3M ESPE, St Paul, MN, USA), and Filtek^™^ Z350 XT flowable composite (3M ESPE, St Paul, MN, USA)
2. Enamel 2: enamel surface treated with Single Bond Universal self-etch adhesive (3M ESPE, Seefeld, Germany) and Filtek Z350 XT flowable composite
3. Enamel 3: enamel surface treated with Scotchbond Universal Etchant and Fusio Liquid Dentin self-adhesive flowable composite (Pentron, Orange CA, USA)
4. Enamel 4: enamel surface treated with Fusio Liquid Dentin self-adhesive flowable composite
5. Dentin 1: dentin surface treated with Scotchbond Universal Etchant, Single Bond Universal Adhesive, and Filtek Z350 XT flowable composite
6. Dentin 2: dentin surface treated with Single Bond Universal self-etch adhesive and Filtek Z350 XT flowable composite
7. Dentin 3: dentin surface treated with Scotchbond Universal Etchant and Fusio Liquid Dentin self-adhesive flowable composite
8. Dentin 4: dentin surface treated with Fusio Liquid Dentin self-adhesive flowable composite

All bonding procedures were performed in accordance with the manufacturers' instructions, as shown in Table 1. After a given surface pretreatment and/or bonding procedure had been performed, the flowable composite was injected into custom-made silicone molds to produce composite posts (length: 2 mm, width: 2 mm). To guarantee that the composite was condensed properly, it was packed in a preset tube with the specific measurement, completely filled, and then condensed further by evenly pressing a glass slab on the surface before curing for 40 s (20 s with the mold in place and another 20 s after it had been removed).

The light-curing procedure was performed using an Elipar^™^ S10 LED Curing Light system (3M ESPE, St Paul, MN, USA) with a light power density of ∼1000 mW/cm^2^; the power density was checked regularly using a Bluephase Meter II radiometer (Ivoclar Vivadent, NY, USA).

All bonded specimens were stored in distilled water at 37°C for 7 days and subjected to 2500 cycles between baths kept at 5 and 55°C using a thermocycling apparatus (Thermocycler 1100/1200, SD Mechatronik, Germany) [20].

A shear bond strength test was performed using a universal testing machine (Instron 5965, MA, USA) with a load cell of 10 kN at a cross-head speed of 0.5 mm/min [20].

After the shear bond strength tests were completed, all fractured specimens were analyzed using a digital microscope (KH-7700, Hirox, NJ, USA) at 50x magnification for failure analysis. The failure modes were labeled according to their area of occurrence: “adhesive failure” if the failure occurred at the tooth surface/composite interface or bonding agent/composite interface, “cohesive failure” if the failure occurred within the composite layer or the tooth structure, and “mixed failure” if the failure was a combination of the above two failure types.

The shear bond strength data were analyzed using the SPSS Statistics (version 21.0) statistical software package. Descriptive statistics (mean and standard deviation) were used to describe the shear bond strength values. One-way analysis of variance and Tukey's test for multiple comparisons were used to compare the mean shear bond strengths for the enamel and dentin surfaces in the eight groups. Also, a two-way analysis of variance was carried out by considering two factors (type of surface and type of treatment) to observe the significance of the interaction between the type of surface and type of treatment on the mean values of shear bond strength. Results with *p* < 0.05 were considered statistically significant.

## 3. Results and Discussion

**Figure 1:**
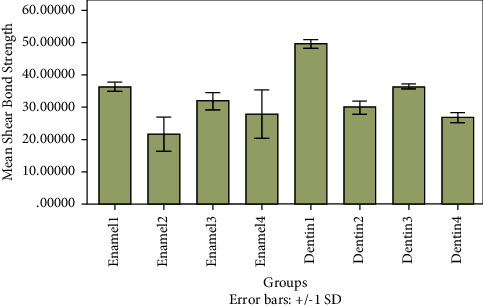
Comparison of mean shear bond strengths of various groups.

A comparison of the mean values of the shear bond strengths for enamel and dentin in the eight groups revealed statistically significant differences (*p* < 0.0001) (Table 2 and Figure 1).

A pairwise comparison of the eight groups indicated that the Dentin 1 group, which was treated with an etch-and-rinse adhesive and Filtek Z350 XT, had the highest mean shear bond strength. By contrast, the Enamel 2 group, which was treated with the Single Bond Universal self-etch adhesive and Filtek Z350 XT, had the lowest bond strength. Furthermore, these differences were highly statistically significant.

The use of the Fusio Liquid Dentin self-adhesive flowable composite on enamel (Enamel 4) and dentin (Dentin 4) resulted in mean bond strengths that were not statistically different. By contrast, the pretreatment of the enamel (Enamel 3) and dentin (Dentin 3) surfaces with a phosphoric acid etch prior to the use of Fusio Liquid Dentin enhanced the bond strength. However, the difference (improvement) in bond strengths was statistically significant only for the dentin groups.

### 3.1. Enamel Groups

**Figure 2:**
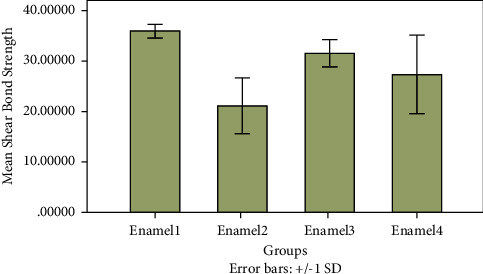
Comparison of mean shear bond strengths of enamel groups subjected to different bonding protocols.

For the enamel groups, the differences in the mean shear bond strengths (*F* = 16.575; *p* < 0.0001) were statistically significant, as shown in Figure 2. A pairwise comparison of the four enamel groups indicated that the group treated with an etch-and-rinse adhesive and Filtek Z350XT (Enamel 1) showed statistically higher mean shear bond strengths than did the groups treated with a self-etch adhesive and Filtek Z350XT (Enamel 2) or the Fusio Liquid Dentin self-adhesive composite alone (Enamel 4). However, the mean bond strength for the Enamel 1 group was not statistically different from that of the group treated with a phosphoric acid etch and the Fusio Liquid Dentin self-adhesive flowable composite (Enamel 3).

With respect to self-etching, the use of the Fusio Liquid Dentin self-adhesive composite alone (Enamel 4) resulted in statistically higher bond strengths as compared to the use of the Single Bond Universal self-etch adhesive (Enamel 2).

Finally, although phosphoric acid pretreatment before the use of the Fusio Liquid Dentin composite (Enamel 3) resulted in higher bond strengths than those when Fusio Liquid Dentin was used without the pretreatment (Enamel 4), the difference was not statistically significant.

**Figure 3:**
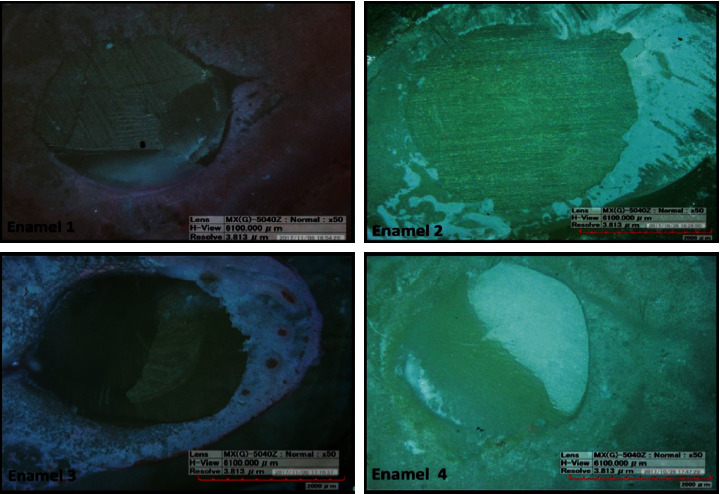
Digital microscopy images of bonded enamel specimens after the shear bond strength test.

An examination of the bonded surfaces of the enamel specimens after the completion of the shear bond strength test showed that all the groups exhibited mixed failure with the exception of the Single Bond Universal self-etch adhesive group (Enamel 2), in which case the complete debonding of the composite and the enamel was observed, with the surface being completely free of an adhesive layer (adhesive failure).  Figure 3 shows images of the failure surfaces.

### 3.2. Dentin Groups

**Figure 4:**
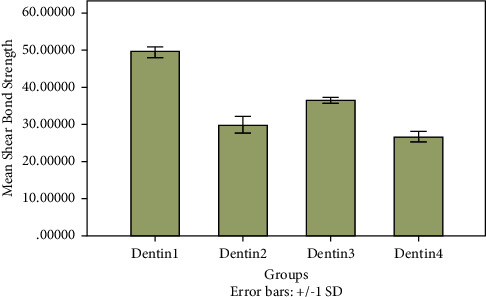
Comparison of mean shear bond strengths of dentin groups subjected to different bonding protocols.

For the dentin groups, the differences in the mean shear bond strengths (*F* = 560.861; *p* < 0.0001) were highly statistically significant. Furthermore, a pairwise comparison showed that the differences between the shear bond strengths were statistically significant (Figure 4). As mentioned earlier, the group treated with an etch-and-rinse adhesive and the Filtek Z350 XT flowable composite (Dentin 1) exhibited statistically higher shear bond strengths than those of all other groups. By contrast, the treatment of dentin with the Fusio Liquid Dentin self-adhesive flowable composite (Dentin 4) resulted in a mean bond strength that was statistically lower than those of the other three groups.

Phosphoric acid pretreatment of the dentin surface before the use of Fusio Liquid Dentin (Dentin 3) resulted in a shear bond strength value that was significantly higher than that of the groups treated with the Single Bond Universal self-etch adhesive (Dentin 2) and with Fusio Liquid Dentin (Dentin 4).

**Figure 5:**
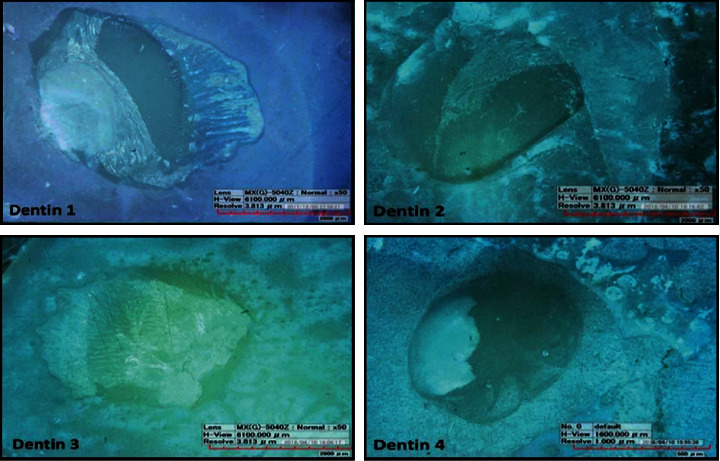
Digital microscopy images of bonded dentin specimens after the shear bond strength test.

Finally, an examination of the bonding surfaces of the dentin specimens after the completion of the shear bond strength test showed that all the groups exhibited mixed failure (Figure 5).

The two-way analysis of variance to compare the shear bond strength between the two surfaces (enamel and dentin) and across the four treatments shows high statistically significant difference for the type of surface (*F* = 59.99, *p* < 0.0001), type of treatment (*F* = 96.16, *p* < 0.0001), and interaction term: Type of surface ^*∗*^ type of treatment(*F* = 14.22, *p* < 0.0001). These results indicate that the mean values of shear bond strength change with the change of surface and with the change of type of treatment.

## 4. Discussion

Flowable composites are now being used widely in clinical practice. In particular, self-adhesive flowable composites enabling the formation of durable bonds through a simple technique have been introduced only recently. Thus, dentists must have adequate knowledge about them so that they can select the most suitable material for a particular application.

In this in vitro study, the shear bond strength of the Fusio Liquid Dentin self-adhesive flowable resin composite (Pentron Clinical, Orange, CA, USA) to enamel and dentin was evaluated. Furthermore, it was assessed whether the pretreatment of enamel and dentin with a phosphoric acid etch affects the shear bond strength of the resin composite and compared its bond strength with those of a self-etch adhesive and an etch-and-rinse adhesive used in conjunction with a conventional flowable composite. A shear bond strength test was employed because it is applicable to bond strength testing of both enamel and dentin and it affords advantages such as small size of the bonded area, simple preparation method for test specimens, and the use of only a small amount of test materials and small number of teeth [19, 20]. The study results obtained for enamel groups suggest that the use of the self-adhesive flowable resin composite results in shear bond strengths that are significantly higher than those of the Single Bond Universal self-etch adhesive group and similar to those for the group where Fusio Liquid Dentin is used after a phosphoric acid pretreatment. Thus, the self-adhesive flowable resin composite is considered promising for use on enamel.

Furthermore, the results of the present study agree with those of a previous study that reported that the bonding effectiveness of flowable self-adhesives in the laboratory is comparable to that of all-in-one adhesives [15]. By contrast, other studies have reported significantly lower bond strengths for self-adhesive flowable composites on enamel as compared to those for conventional flowable composites bonded using etch-and-rinse adhesives [21, 22]. Self-adhesive flowable resin composites result in significantly higher bond strengths on enamel when the surface is pretreated with a phosphoric acid etch and/or an adhesive bonding agent [17]. Mine et al. [23] suggested a probable explanation for the lower bond strength of self-adhesive composites; they used transmission electron microscopy to show that limited interactions occur between the smear-covered substrates and the aprismatic enamel. This explains why some studies have reported that the bond strength of self-adhesive composites is poorer than that of total-etch adhesives.

The lowest mean shear bond strength was seen for the enamel samples treated with the self-etch universal adhesive and the flowable composite. In this case, the shear bond strength was significantly affected by the pretreatment with phosphoric acid. This finding agrees with the results of several previous studies and confirms the importance of using an acid etch surface pretreatment with self-etch adhesive systems, particularly for enamel [14, 24–28]. The decreased bond strength of the self-etch adhesive on enamel can be explained based on these previous studies. The acidic monomers present in self-etch adhesives can partially dissolve the smear layer and etch the enamel. However, even the strongest self-etching systems are not as acidic as phosphoric acid. The phosphoric acid pretreatment likely increases the roughness of the enamel surface and removes the superficial enamel layer, making the enamel more receptive to the self-etching system. In addition to the etching pattern, the bonding resin should be able to penetrate through the microspaces created by the phosphoric acid or acidic monomers to produce a highly cross-linked polymer when subjected to light curing. This consequently leads to the formation of long resin tags and, in turn, deeper penetration of the enamel adhesive [14, 26–28].

For dentin groups, the shear bond strength test results indicated that the dentin group treated with an etch-and-rinse adhesive system and a conventional flowable nanocomposite had the highest mean shear bond strength among all eight experimental groups. These findings agree with previously published data that indicated that acid etching enhances the bond strength even on smear-free dentin surfaces [26, 29].

Unlike in the case of enamel samples, the use of the Fusio Liquid Dentin self-adhesive flowable resin composite on dentin not subjected to a surface pretreatment resulted in the lowest shear bond strength among all dentin groups. These results agree with those of previous studies that reported that self-adhesive flowable composite formulations on dentin surfaces have low bond strengths [21, 30–32]. This data can be interpreted based on previously published information on self-etching adhesives with high pH values. The low bond strength of self-etch adhesives with high pH may be related to their inability to etch superficial dentin. It may also be related to their hydrophilic nature; these adhesives attract water that is not evaporated readily and gets trapped and that subsequently diffuses back rapidly from the bonded dentin to the adhesive resin and lowers the strength of the mechanical bond [32].

Finally, the shear bond strengths of the Fusio Liquid Dentin resin composite on enamel and dentin were comparable. In addition, the self-adhesive composite performed as good as the self-etch adhesive but had lower bond strength value when compared to etch-and-rinse adhesive system. Phosphoric acid pretreatment is essential for achieving high shear bond strength for both enamel and dentin.

Based on the abovementioned results, both null hypotheses were rejected.

The results of this study are in line with previous publications on self-adhesive flowable composites. The limitations of this study include the number of materials tested, as the use of different self-adhesive flowable composites may provide more accurate details on this class of materials. Also, while the use of shear bond strength test is simple, it has been suggested that the conduction of shear bond strength test may be more efficient in studying the complex interaction between composite and substrate [33]. Finally, this is an in vitro study in a lab setting, and further in vivo studies could be employed to compare the actual clinical success of such materials.

Further investigations are needed to confirm the results of the present study and to evaluate the performance of other self-adhesive flowable composites in lab and clinical settings.

## 5. Conclusions

Within the limitations of the present study, the Fusio Liquid Dentin self-adhesive flowable resin composite is concluded to exhibit similar shear bond strengths on enamel and dentin and the bond strengths were lower than those of conventional flowable composites used in combination with etch-and-rinse adhesives. Phosphoric acid etching pretreatment improved the bond strength of self-adhesive flowable composites.

## Figures and Tables

**Table 1:** Materials used in this study.

Material	Manufacturer	Material composition	Application procedure
Fusio^™^ Liquid Dentin self-adhesive flowable resin composite	Pentron, Orange, CA, USA	4-META (4-methacryloxyethyl trimellitic acid)-based flowable composite containing nanosized amorphous silica	(1) Apply composite in 1 mm increments and agitate for 20 s (2) Light cure for 10 s (3) Apply composite in 2 mm increments and light cure for 20 s

Filtek^™^*Z* 350 XT Flowable composite	3M ESPE Dental Products, St. Paul, MN, USA	Bis-GMA (bisphenol A-glycidyl methacrylate), Bis-EMA (bisphenol a diglycidyl methacrylate ethoxylated), UDMA (urethane dimethacrylate), zirconia/silica (78% w/w), barium glass, ytterbium trifluoride, mixed oxide prepolymer	(1) Apply composite in 2 mm increments and light cure for 20 s

Single Bond Universal Adhesive	3M ESPE, Seefeld, Germany	MDP phosphate monomer, dimethacrylate resins, HEMA (2-hydroxylethyl methacrylate), Vitrebond copolymer, filler, ethanol, water, initiators, silane	If used as total-etch adhesive: (1) Etch enamel with phosphoric acid for 15 s, rinse, and air dry (2) Apply adhesive, air dry for 5 s, and light cure for 10 s If used as self-etch adhesive: (1) Apply adhesive coat and agitate for 20 s (2) Air dry for 5 s and light cure for 10 s

Scotchbond™ Universal Etchant	3M ESPE Dental Products, St. Paul, MN, USA	32 wt% phosphoric acid etching gel	(1) Etch enamel or dentin surface for 15 s, wash, and air-dry

**Table 2:** Comparison of mean shear bond strengths of eight enamel and dentin groups.

Test group	Mean (standard deviation)	F value	*P* value
Enamel 1	36.19 (1.40)	55.877	<0.0001
Enamel 2	21.41 (5.27)		
Enamel 3	31.79 (2.51)		
Enamel 4	27.69 (7.69)		
Dentin 1	49.38 (1.23)		
Dentin 2	29.68 (1.90)		
Dentin 3	36.25 (0.60)		
Dentin 4	26.58 (1.33)		

## Data Availability

Data are available through the supplementary information file submitted with the manuscript.

## Supplementary Materials

Supplementary Materials Study results of shear bond strength data for all test groups are included as supplementary materials.
