# Supplementary material for: Shear Bond Strength of Self-Adhesive Flowable Resin Composite
Source: Int J Dent. 2022 May 6;2022:6280624. doi: 10.1155/2022/6280624 (PMC9106484; doi:10.1155/2022/6280624)
Supplement: Supplementary Materials — Study results of shear bond strength data for all test groups are included as supplementary materials. [file 6280624.f1.docx]

**Shear Bond Strength Values - Study Results**

**Group 1 = Enamel 1**

| Group E1 - Specimen | Shear Bond Strength Value |
| --- | --- |
| 1 | 34.82235 |
| 2 | 34.94880 |
| 3 | 35.08228 |
| 4 | 38.97021 |
| 5 | 35.41129 |
| 6 | 37.86177 |
| 7 | 36.88030 |
| 8 | 35.67623 |
| 9 | 35.31243 |
| 10 | 36.89499 |

**Group 2 = Enamel 2**

| Group E2 - Specimen | Shear Bond Strength Value |
| --- | --- |
| 1 | 15.64429 |
| 2 | 21.01056 |
| 3 | 30.48895 |
| 4 | 29.84308 |
| 5 | 20.00555 |
| 6 | 18.37646 |
| 7 | 16.45456 |
| 8 | 24.89884 |
| 9 | 19.33265 |
| 10 | 18.02961 |

**Group 3 = Enamel 3**

| Group E3 - Specimen | Shear Bond Strength Value |
| --- | --- |
| 1 | 36.84580 |
| 2 | 34.33992 |
| 3 | 31.80452 |
| 4 | 33.57639 |
| 5 | 28.47484 |
| 6 | 29.49335 |
| 7 | 31.35608 |
| 8 | 29.95383 |
| 9 | 30.60590 |
| 10 | 31.46952 |

**Group 4 = Enamel 4**

| Group E4 - Specimen | Shear Bond Strength Value |
| --- | --- |
| 1 | 6.33320 |
| 2 | 32.97308 |
| 3 | 28.18188 |
| 4 | 30.29972 |
| 5 | 31.60828 |
| 6 | 31.58422 |
| 7 | 30.29601 |
| 8 | 28.40733 |
| 9 | 29.69763 |
| 10 | 27.49295 |

**Group 5 = Dentin 1**

| Group D5 - Specimen | Shear Bond Strength Value |
| --- | --- |
| 1 | 51.63107 |
| 2 | 47.68655 |
| 3 | 47.99795 |
| 4 | 48.87633 |
| 5 | 50.69846 |
| 6 | 49.78150 |
| 7 | 48.71610 |
| 8 | 50.14050 |
| 9 | 49.57231 |
| 10 | 48.57307 |

**Group 6 = Dentin 2**

| Group D6 - Specimen | Shear Bond Strength Value |
| --- | --- |
| 1 | 30.33985 |
| 2 | 28.43899 |
| 3 | 25.64126 |
| 4 | 30.07885 |
| 5 | 32.44594 |
| 6 | 29.55355 |
| 7 | 28.29805 |
| 8 | 31.53748 |
| 9 | 30.68879 |
| 10 | 29.81933 |

**Group 7 = Dentin 3**

| Group D7 - Specimen | Shear Bond Strength Value |
| --- | --- |
| 1 | 36.01234 |
| 2 | 35.40004 |
| 3 | 36.15492 |
| 4 | 36.82184 |
| 5 | 36.93732 |
| 6 | 35.85237 |
| 7 | 36.89627 |
| 8 | 35.59157 |
| 9 | 35.92070 |
| 10 | 36.92017 |

**Group 8 = Dentin 4**

| Group D8 - Specimen | Shear Bond Strength Value |
| --- | --- |
| 1 | 27.09946 |
| 2 | 27.49984 |
| 3 | 25.59416 |
| 4 | 26.79261 |
| 5 | 25.85418 |
| 6 | 24.93817 |
| 7 | 25.31419 |
| 8 | 25.57223 |
| 9 | 28.86285 |
| 10 | 28.30105 |
